# A case of acute promyelocytic leukemia variant with derivative chromosome 3 der(3)t(3;8) associated with 8q partial gain

**DOI:** 10.1186/s13039-019-0445-1

**Published:** 2019-07-05

**Authors:** Filomena Nozza, Gabriella Vona, Stefania Trino, Fiorella D’Auria, Francesco La Rocca, Vitina Grieco, Luciana Possidente, Luciana De Luca, Pellegrino Musto

**Affiliations:** 1 Laboratory of Clinical Research and Advanced Diagnostics, IRCCS-CROB, Referral Cancer Center of Basilicata, Via Padre Pio 1, 85028 Rionero in Vulture, PZ Italy; 2 Laboratory of Preclinical and Translational Research, IRCCS-CROB, Referral Cancer Center of Basilicata, Via Padre Pio 1, 85028 Rionero in Vulture, PZ Italy; 3 Unit of Hematology and Stem Cell Transplantation and Hematology Department of Basilicata, IRCCS-CROB, Referral Cancer Center of Basilicata, Via Padre Pio 1, 85028 Rionero in Vulture, PZ Italy

**Keywords:** Acute promyelocytic leukemia, Molecular cytogenetics, *c-myc* gene, FISH, Painting, Chromosome 8

## Abstract

**Background:**

Acute promyelocytic leukemia (APL) is characterized by fusion of PML/RARα genes as a result of t(15;17)(q24;q21). APL is now one of the curable hematological malignancies thanks to molecularly targeted therapies based on all-trans retinoic acid (ATRA) and arsenic trioxide (ATX). Extramedullary (EM) relapse is a rare event in APL, ear involvement being even more infrequent, with only six cases so far described. About 30–35% of patients with newly diagnosed APL have additional cytogenetics abnormalities, whose prognostic significance is still controversial. The most common additional aberration is trisomy 8 or partial gain 8q.

**Case presentation:**

We describe here a novel unbalanced translocation der(3)t(3;8)(q29;q23.3-q24.3) associated with 8q partial gain in a 41 year-old man affected by APL in molecular remission after first line treatment, who had a responsive EM relapse in the auditory canal.

**Conclusions:**

EM relapse is a rare event in APL and ear involvement is even more infrequent. To our knowledge, this is the first reported case of APL with a new der(3)t(3;8)(q29;q23.3-q24.3) and 8q partial gain associated with t(15;17)(q24;q21). Despite the recurrence of the disease at EM level, the clinical outcome of this patients was favorable.

## Background

Acute Promyelocytic Leukemia (APL) is a subtype of Acute Myeloid Leukemia (AML), characterized by fusion of Promyelocytic Leukemia (PML) and Retinoic Acid Receptor Alpha (RARα) genes as a result of t(15;17)(q24.1;q21.2), which can be seen in up to 90% of APL cases [1]; in a minority of patients, it may be cryptic or results from complex cytogenetic rearrangements other than t(15;17) [2]. Cryptic t(15;17) on i(17q), in particular, leads to an extra copy of PML/RARα, which may confer a worse prognosis [3]. Currently, APL is one of the most curable hematological malignancies, due to tailored chemotherapy and molecularly targeted treatments based on all-trans retinoic acid (ATRA) and arsenic trioxide (ATX) [4, 5].

About 30–35% of patients with newly diagnosed APL harbor additional cytogenetics abnormalities, whose prognostic significance is still controversial [6]. In APL the most common additional aberration is trisomy 8 or partial gain 8q, which could induce a *c-myc* gene dosage effect [7, 8].

EM localizations are a quite rare event in patients with APL [9–13], the most common sites being skin [14] and central nervous system, particularly in pediatric cases [15–17]. Among the EM sites, ear involvement is infrequent and small case series have been described [18–22].

We report here an original APL case in which along with t (15;17), a new additional chromosomal abnormality, der(3)t(3;8)(q29;q23.3-q24.3), associated with a 8q gain, is described in a patient who developed an isolated EM relapse of the ear during the course of his disease.

## Case presentation

A 41-year old man was admitted to our Institute on July, 2014, because of a white blood cell count (WBC) of 100 × 10^9^/l, a hemoglobin level of 116 g/l and a platelet count of 82 × 10^9^/l. Cytological analysis of peripheral blood and bone marrow (BM) aspirate led to a morphological possible diagnosis of hypo-granular APL. The immuno-phenotype was positive for CD2, CD64, CD56, CD13, CD33, MPO, CD38. Cytogenetic analysis and nested PCR confirmed the presence of PML/RARα fusion gene (BCR3), which was also detected by using fluorescence in situ hybridization (FISH). Based on morphological, phenotypic, molecular and cytogenetic findings, the patient was diagnosed as having an APL “variant” and treated with AIDA 2000 protocol (ATRA and Idarubicin). After hematological and molecular complete remission (CR) were obtained, three following consolidation cycles were administered. On February 2015, however, the patient developed an isolated symptomatic EM relapse in the auditory canal. The patient received FLAG regimen (fludarabine, high-dose cytarabine and granulocyte-colony stimulating factor G-CSF), obtaining a new CR, that is currently maintained, 4 years after the first diagnosis.

## Methods

Cytogenetic analysis at diagnosis was carried out according to a standard procedure on 24-h cultured BM cells. G-banded chromosome were identified according to International System for Human Cytogenetic Nomenclature (ISCN 2016). FISH analysis was performed according to the manufacturer’s instructions on fixed nuclei using commercially available PML/RARα dual color dual fusion DNA probe, C-MYC Break Apart Rearrangement Probe Kit, LSI CEP8 Spectrum Orange Direct Labeled Fluorescent DNA Probe KIT, RPN1/MECOM DF FISH Probe Kit (Vysis Abbott Molecular Inc., IL USA), BCL6 FISH DNA Probe Split Signal (Dako Denmark A/S) and a whole chromosomes 8 and 3 painting probes (WCP8 and WCP3) (Cytocell Ltd. 3–4 Technopark Newmarket Road, Cambridge). FISH was also used to evaluate the presence of fusion PML/RARAα on the biopsy specimen of the ear mass (paraffin tissue). Fluorescent signals were visualized with Nikon microscope with double filter and at least 200 interphase cells were scored for signal patterns.

Chromosomal Microarray Analysis (CMA) was performed by using Infinium CytoSNP-850 K (Illumina San Diego, CA, USA) according to the manufacturer’s instructions. The array contains approximately 850.000 single nucleotide polymorphisms (SNPs) markers spanning the entire genome with an average probe spacing of 1,8 Kb. The data were analyzed using BlueFuse Multi software v4.2 and GenomeStudio Data Analysis Software v. 2010 based on the reference human genome (hg19/GRCh37).

## Results

**Fig. 1:**
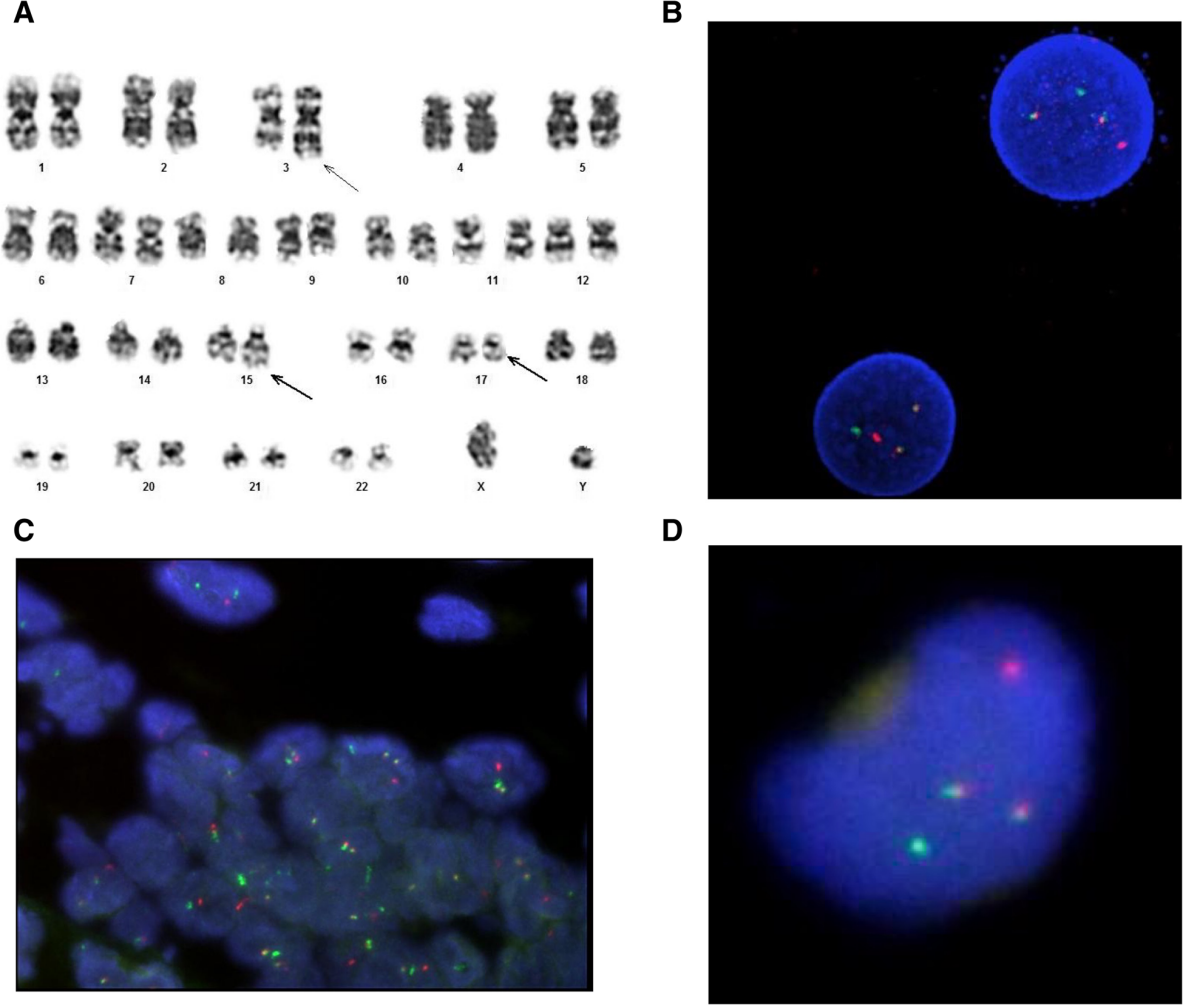
**a** Abnormal karyotype detected by G-banding, showing der(3)t(3;8) and t(15;17)(q24;q21) (arrows), **b** FISH analysis showing PML/RARA rearrangement (green, red and two yellow fusion signals) on BM at diagnosis; **c** FISH analysis showing PML/RARA rearrangement (green, red and two yellow fusion signals) on ear mass biopsy (paraffin tissue); **d** the same sample of C at higher magnification

At diagnosis, BM G-banding showed the following karyotype: 46,XY,t(15;17)(q24;q21),der(3)t(3;?)(q?;?) or add(3)(q?) in 20 metaphases (Fig. 1a). FISH confirmed the presence of the PML/RARα fusion gene (Fig. 1b).

At time of EM relapse, BM karyotype was 46,XY and both FISH and quantitative PCR analyses were negative for PML/RARα fusion gene. Conversely, FISH analysis, performed on the biopsy specimen of the ear mass (paraffin tissue) using LSI PML/RARα dual color dual fusion probe (Vysis), revealed t(15;17) on 100% of examined cells (Fig. 1c and d).

**Fig. 2:**
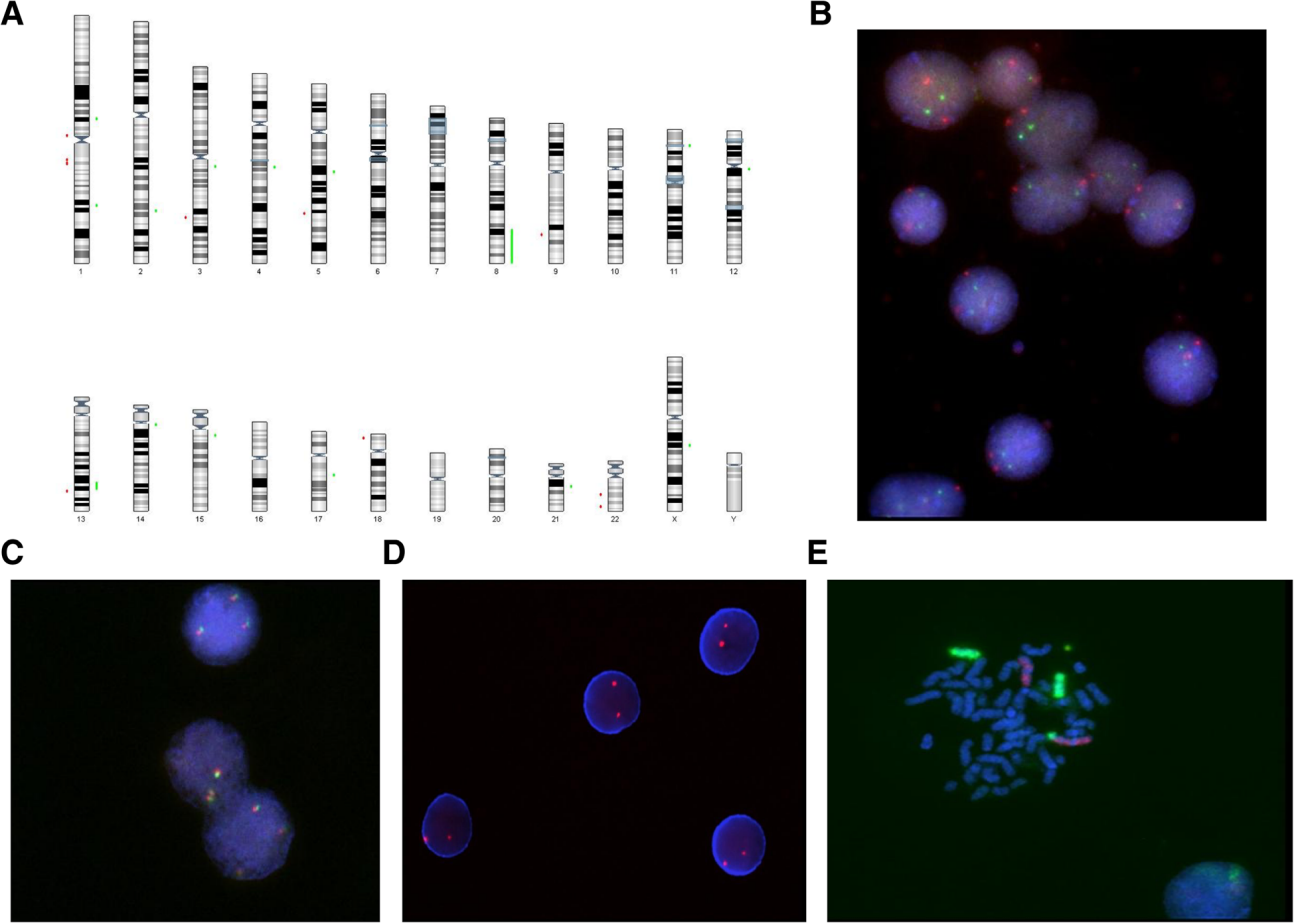
**a** Karyogram according to SNP-A analysis. Gain appears in green at the right of chromosome 8; **b** FISH analysis with RPN1/MECOM DF probe showing a normal pattern of hybridization on BM at diagnosis; **c** FISH analysis with MYC break-apart probe showing three *c-myc* alleles without breaking on BM at diagnosis; **d** FISH analysis with the centromere (CEP 8) probe on BM cells at diagnosis showing a normal pattern of hybridization; **e** characterization of der(3) by FISH with whole chromosome painting 3 (red) and 8 (green) on BM cells at diagnosis

To further characterize the nature of extra-material on the long arm of chromosome 3, identified by karyotype, we performed CMA on BM at diagnosis observing gain and loss alterations in different chromosomes. In particular, chromosome 3 displayed a gain of 3q12.2 and loss of 3q25.1, but no gain or loss were observed in the region including 3q25.1-3q29 (Fig. 2a). However, we performed FISH on BM to evaluate a possible rearrangement of EVI1 (Fig. 2b) and BCL6 (data not shown) located respectively in q26 and q27 bands, observing a normal pattern of hybridization. Of note, CMA analysis showed a gain of long arm of chromosome 8q23.3–24.3 (Fig. 2a), that could explain the nature of extra-material on chromosome 3. To validate array data, we studied *c-myc* gene by FISH on BM at diagnosis, mapping in 8q24.21 and included the gained region. Using double color FISH Break Apart rearrangement probe for *c-myc* gene and CEP8 Spectrum Orange Direct Labeled Fluorescent DNA Probe KIT, we observed three *c-myc* alleles without breaking (Fig. 2c), but a normal pattern of hybridization for the centromeric region, confirming CMA data (Fig. 2d). Two-color FISH was also performed on BM at diagnosis with painting probes for whole chromosome 8 and 3 altered by karyotype and array respectively (Cytocell Ltd. 3–4 Technopark Newmarket Road, Cambridge). Interestingly, this analysis confirmed the additional abnormality der (3) t(3;8)(q29;8q23.3-q24.3). Thus, the karyotype with microarray nomenclature was: 46,XY,t(15;17)(q24;q21),der(3)t(3;8)(q29;q23.3–24.3).arr8q23.3q24.21(112,491,668- 146,293,414)×3″ (Fig. 2e).

## Discussion and conclusions

Gain of chromosome 8 is the most common and frequent chromosomal alteration in AML, including APL [23]. In general, it is found not only at diagnosis, as an apparently primary event, but also during disease progression, as a secondary chromosome change involved in unbalanced translocation [24]. Patients with +8 as the sole cytogenetic abnormality have intermediate prognosis, while patients with +8 in addition to favorable chromosome aberrations maintain a good clinical outcome [25].

We report here, to the best of our knowledge, the first case of a newly diagnosed APL and EM ear relapse with der (3)t(3;8)(q29;8q23.3-q24.3) associated with a partial 8q gain, and t(15;17)(q24;q21). Literature data indicate that t(3;8) has been previously described in hematological malignancies [26–28]; in particular, the rearrangement of 3q26 and 3q27 bands, which disrupt respectively EVI1 and BCL6, may also occur in a variety of reciprocal translocations which have been reported in myelodysplastic syndrome, AML and lymphoma [26]. Of note, in our case we didn’t find any rearrangement of EVI1 and BCL6. Moreover, painting analysis indicated that chromosome 3 displayed no abnormalities and the gain of long arm of chromosome 8 was banded at the end of chromosome 3. Thus, we report a new alteration involving chromosome 3 with gain of long arm of chromosome 8, which has not been previously described. It’s well established that 8q23–24 region plays a role in leukemogenesis. In fact, this region includes many genes involved in cell growth regulation, differentiation and apoptosis. One of the possible candidate genes is *c-myc* oncogene, which is overexpressed in different human tumors [8, 29]. Our result supports, as in other AML, the possible pathogenic importance of this region also in APL and our finding of der(3)t(3;8)(q29;8q23.3-q24.3) is in agreement with the *c-myc* gene dosage theory. The involvement of long arm of chromosome 3, however, suggests that more than one gene could be altered. The better understanding of the DNA structure of this region and the identification of other relevant genes could provide further insight into their potential role in leukemia.

EM disease at presentation or at relapse is considered an unusual event in APL [13]. Of note, in the last 20 years, in which ATRA has become an integral part of APL treatment, the number of reported EM relapse in APL patients seems to be increased [11, 22]. Interestingly, EM relapse has been associated to high WBC count at diagnosis, BCR3 isoform of PML/RARα and micro-granular variant [13], all findings present in our case. Finally, achievement of a second CR, even after EM relapse, in our patient is in agreement with the previously reported observation that the presence of other additional abnormalities, such as der(3)t(3;8)(q29;8q23.3-q24.3), does not necessarily worsen the favorable prognosis of patients harboring isolated t(15;17).

## Data Availability

The data sets used and/or analyzed during the current study are available from the corresponding author on reasonable request. All authors read and approved the final manuscript.

## Abbreviations

AML: Acute Myeloid Leukemia
APL: Acute promyelocytic leukemia
ATRA: All-trans retinoic acid
ATX: arsenic trioxide
BM: bone marrow
CMA: Chromosomal Microarray Analysis
CR: Complete remission
EM: Extramedullary
FISH: Fluorescence in situ hybridization
SNPs: Single nucleotide polymorphisms
WBC: White blood cell count
